# Surgery of the Pancreas and Spleen

**Published:** 1904-07-09

**Authors:** 


### SURGERY OF THE PANCREAS AND SPLEEN.

**Pancreatic Surgery**, says Mikulitz,[1] has undergone a tardy development, for the following reasons: 1. The topographical relations of the organ—its hidden and protected position. 2. The difficulty of diagnosis; the main obstacle here again being its concealed position. Experience has shown that in affections of the pancreas, which have been treated surgically, positive functional disturbances have been observed only in rare instances. 3. The operation, so far as it includes the organ itself, is much more dangerous than an operation upon any other abdominal organ. This is partly the result of the low condition of most patients suffering from pancreatic disease, while a further danger lies in the peculiar physiological character of the gland itself. The escape of pancreatic juice seriously affects the peritoneum and neighbouring tissues. The secretion from the injured pancreas leaking into the abdominal cavity can of itself so damage the peritoneum, that death from this cause alone may result. The pancreatic juice mixed with blood has, no doubt, a very toxic effect, and can, in the so-called apoplexy of the pancreas, result fatally without the complication of bacterial infection. In this connection the author does not refer to the normal physiological secretion, but rather to the exudate from the injured region. Whatever operation may be done on the pancreas, we must take the greatest pains to prevent the secretion of the diseased gland from getting into the abdominal cavity. This may be done in two ways—either by turning the injured part inwards and closing it with deep sutures, so that the peritoneal covering is again in continuity, or by the use of the tampon. Robson[2] says we may derive assistance in the diagnosis of pancreatic disease if we bear in mind the following conclusions:—1. Fat occurs in the stools in three forms: (*a*) as fat droplets; (*b*) as fatty acid crystals; and (*c*) as soap crystals. 2. The capacity for digestion and absorption of fats is limited; if, therefore, fat be taken in large quantities, it is found in the stools. 3. Steatorrhœa occurs in some cases of jaundice, in some cases of enteritis, and in some affections of the pancreas, but in none of these constantly. 4. When jaundice and interstitial pancreatitis co-exist, there is a great excess of fat in the stools. 5. The presence of an excess of fat in the motions in the absence of jaundice and diseases of the intestine is suggestive of pancreatic disease. 6. If the pancreatic reaction (described later) be found in the urine along with steatorrhœa, some affection of the pancreas is almost certain. 7. If azotorrhœa be found along with steatorrhœa, it is almost certain that the pancreas is diseased, and if the pancreatic reaction in the urine, diabetes, and an epigastric tumour be present the diagnosis is almost certain. Besides the local hæmorrhage that may be known as pancreatic apoplexy and the form known as hæmorrhagic pancreatitis, a tendency to general hæmorrhage from mucous surfaces, and in and under the skin exists in many pancreatic affections. As to the chemical changes, it is well known that a diminution in the lime salts leads to a tendency to hæmorrhage, and as he has found that there is in nearly every case of pancreatitis a profuse excretion of lime in the shape of oxalates, this gives another explanation, and also explains the beneficial effects resulting from the administration of calcium chloride. Diabetes is only present when the islands of Langerhans are involved, as in the interacinar type of chronic interstitial pancreatitis. Fat necrosis is commonly found in association with pancreatitis and other diseases of the pancreas. It is always the result of the penetration of the fat-splitting ferment of the pancreas first into the tissues in the neighbourhood of the gland and when more extensive to the diffusion of the ferment either through continuity of tissue or by means of the lymphatics. It is associated with a splitting up of the fat into fatty acids and glycerine; the latter is absorbed, but the acids being insoluble remain in the cells either as crystals or uniting with the calcium salts of the blood they form yellowish-white patches of various size. If iodoform be enclosed in a gelatine capsule, hardened by formalin, and given by the mouth, it is almost unaffected by gastric digestion, but is readily dissolved by the pancreatic secretion. If pancreatic digestion is normal, iodine should appear in the urine in from four to eight hours. The absence of the reaction or its delayed appearance, if the motor function of the stomach be normal, indicates, according to Sahli, an impairment of pancreatic digestion. The pancreatic test referred to above depends upon the presence of glycerine in the manner already described. Cammidge[3] describes the means of detecting this in the urine by the formation of glycerose when a solution of glycerine is treated with nitric acid. He uses phenylhydrazin as the means for detecting the glycerose, and says that an exceedingly well-marked deposit of crystals is given by pancreatic urines. By noting the character of the crystals and their rate of solubility in dilute sulphuric acid he claims to be able to differentiate between chronic inflammation and malignant disease of the pancreas.

**Inflammatory Affections of the Pancreas**, says Robson,[4] may be catarrhal, the inflammatory trouble being in the ducts, or parenchymatous, the substance of the pancreas being involved. Catarrhal inflammations include :—(*a*) Simple catarrh, acute, and chronic. (*b*) Suppurative catarrh. Parenchymatous inflammation include :—I. Acute, (*a*) Hæmorrhagic pancreatitis. 1. Ultra-acute, in which the hæmorrhage precedes the inflammation, the bleeding being profuse, and both within and outside the gland. 2. Acute, in which inflammation precedes the hæmorrhage, which is less profuse and is distributed in patches through the gland. (*b*) Gangrenous pancreatitis. (*c*) Suppurative pancreatitis (diffuse suppuration). II. Subacute. Abscess of the pancreas (not diffuse suppuration). III. Chronic. (*a*) Interstitial pancreatitis. 1. Interlobular. 2. Interacinar. (*b*) Cirrhosis of pancreas. The etiology of pancreatitis may be classified under predisposing and exciting causes. Predisposing causes :—(*a*) Obstruction in the ducts, the result of gall-stones, duodenal catarrh, pancreatic calculi, cancer of the papilla or of the head of the pancreas, ulcer of the duodenum, followed by cicatricial stenosis of the papillæ, ascarides, and lumbrici. (*b*) Injury either from a bruise, or by manipulation in operating, or from a crush, or by a blow in the epigastrium, or from wounding by a sharp instrument. (*c*) Hæmorrhage into the gland. (*d*) General ailments, such as typhoid fever, influenza, or mumps. (*e*) Certain anatomical peculiarities in the pancreas or its ducts. (*f*) Atheroma or fatty degeneration of blood-vessels. Exciting causes :—1. Infection, conveyed either—(*a*) From the blood, as in syphilis or pyæmia. (*b*) From the duodenum, as in gall-stone obstruction or gastro-intestinal catarrh. (*c*) By extension inwards from adjoining organs, as in gastric ulcer or cancer eroding the pancreas. (2) Irritation, as in alcoholism (?) Gall-stones in the common duct are frequent—in fact, by far the most frequent—cause of the various forms of pancreatitis, but acute pancreatitis may occur apart from cholelithiasis. Acute pancreatitis is to be suspected when a previously healthy person or sufferer from occasional attacks of indigestion is suddenly seized with violent pain in the epigastrium, followed by vomiting and collapse, and, in the course of 24 hours, by a circumscribed epigastric swelling, tympanitic or resistant, with slight rise of temperature.

**Pancreatic Cysts.**—Cumston[5] says that little is known about pancreatic cysts. He accepts the classification of Kört :—(1) Retention cysts from obstruction of the excretory duct. (2) Proliferation cysts of the pancreatic tissue. (3) Retention cysts, originating from the glandular vesicles and smaller excretory ducts, the result of chronic interstitial pancreatitis. (4) Pseudo-cysts, which arise from inflammatory or traumatic lesions. Robson and Moynihan add congenital cystic disease and hydatid cysts, which latter are also found in other classifications. They include, under the head of proliferation cysts, crystadenoma and cystic epithelioma. The direction of the fistula now points to a part of the pancreas nearer the tail than the head. The more common location of these cysts is above the transverse colon. Robson,[6] who also deals with this subject, points out the sufficiency of simple incision and drainage. The mortality after complete, or even partial, extirpation has been high.

**Cysts of the Spleen.**—Heinricus[7] adopts Litten's classification :—(1) Blood cysts or lymphatic cysts (either uni- or multi-locular) non-parasitic in origin. (2) Hydatid cysts (the most common variety). (3) Dermoid cysts (rare). All these different varieties are clinically indistinguishable, and the diagnosis of them must always be extremely difficult and generally can only be made either by operation or post-mortem examination. The various methods of treatment which have been applied are described, namely, simple puncture, puncture and injection of iodine, splenotomy and drainage, extirpation of the cyst, and splenectomy. Of all these methods, splenectomy is the only one to be advised.

1 Dublin Journ. of the Med. Sci., November 1903, p. 367. 2 Lancet, March 19th, 1904, p. 775. 3 Lancet, March 19th, 1904, p. 782. 4 Brit. Med. Journ., March 26th, 1904. 5 Annals of Surgery, March 1904, p. 456. 6 Brit. Med. Journ., April 2nd, 1904, p. 773. 7 Med. Chron., February 1904, p. 342.

